# Comparative analysis of morning and evening training on performance and well‐being in elite soccer players

**DOI:** 10.14814/phy2.70510

**Published:** 2025-08-11

**Authors:** Okba Selmi, Mohamed Amine Rahmoune, Anissa Bouassida, Santo Marsigliante, Antonella Muscella

**Affiliations:** ^1^ High Institute of Sports and Physical Education of Kef University of Jendouba El Kef Tunisia; ^2^ Research Unit Sportive Sciences, Health and Movement El Kef Tunisia; ^3^ Department of Biological and Environmental Sciences and Technologies (Di.S.Te.B.A.) Lecce Italy

**Keywords:** circadian rhythms, mood states, perceived exertion, small‐sided games, soccer performance, time of day

## Abstract

The purpose of the study was to determine how training at different times of day affects soccer players' performance, mood, and physiological responses. Twenty male professional soccer players participated in the study, which involved small‐sided games (SSGs) conducted in both morning and evening sessions. Players' mood states, perceived exertion, heart rate, and performance metrics were measured to assess the impact of circadian rhythms on their physical and psychological responses. Results showed significantly higher HRmean (*p* = 0.025) and %HRmax (*p* = 0.022) and significantly lower blood lactate concentration (*p* = 0.015) in the morning. Despite similar perceived exertion and recovery scores, players reported greater enjoyment in evening sessions (*p* = 0.0008). Mood states were more favorable in the evening, with significantly lower levels of stress (*p* = 0.007), fatigue (*p* = 0.009), and muscle soreness (*p* = 0.012). The Hooper's Index was also markedly lower in the evening (*p* = 0.0008). Evening performance was superior, with significantly more ball interceptions (*p* = 0.033), successful passes (*p* = 0.0004), and ball possession recoveries (*p* = 0.025), alongside fewer lost balls in the evening compared to the morning (*p* = 0.008). In conclusion, the study demonstrates that time of day significantly influences both psychological and physical responses in professional soccer players, with evening sessions leading to a more favorable mood and better performance. These findings support the consideration of circadian rhythms when scheduling training and matches to optimize athletic outcomes.

## 1 INTRODUCTION

In competitive sports, an athlete's performance is influenced by various factors, including physiological, physical, technical, tactical, and cognitive skills.

During exercise, several biochemical and physiological processes and psychological responses can be affected by the time of day, reflecting individual differences in circadian timing (Chtourou & Souissi, 2012; Grgic et al., 2019; Lack et al., 2009).

Circadian rhythms are biological processes that follow a cycle of approximately 24 h, influencing various physiological functions such as core body temperature, heart rate (HR), blood pressure, and many hormones (Gnocchi & Bruscalupi, 2017), and psychological factors. These rhythms are regulated by the suprachiasmatic nucleus in the brain and are influenced by external factors such as light exposure, sleep, and temperature (Refinetti, 2020).

Circadian rhythms, while influenced by age, daylight hours, sleep patterns, or type of exercise, remain relatively stable overall (Reid, 2019). Furthermore, it has been shown that circadian rhythms not only regulate key physiological processes involved in athletic performance (Chtourou, Chaouachi, Hammouda, et al., 2012; Chtourou & Souissi, 2012; Hower et al., 2018), but also that disruption of these rhythms can negatively impact both physical and cognitive performance (Atkinson & Reilly, 1996; Wolff & Esser, 2019). For example, body temperature peaking in the late afternoon is associated with increased muscle strength, reaction time, coordination, cardiovascular efficiency, and overall performance (Anderson et al., 2018; Kunorozva et al., 2014; Küüsmaa et al., 2016; Shibata & Tahara, 2014; Starkie et al., 1999). Additionally, maximal anaerobic power and flexibility are also higher in the late afternoon, correlating with increased temperature (Racinais et al., 2005). This has led to the recommendation that training programs and competitions should ideally align with these peaks to maximize performance outcomes (Ayala et al., 2021).

Cortisol levels follow a circadian pattern; in fact, in resting conditions, they reach their peak in the morning and then gradually decrease during the day. Cortisol is involved in preparing the body for physical activity by increasing the energy available for exercise, and during physical activity, cortisol levels increase in the morning and then decrease throughout the day (De Nys et al., 2022). Then the timing of exercise could influence the cortisol response and, consequently, physical performance. Plasma catecholamines also exhibit circadian rhythms at rest, although their patterns during exercise may vary (Brito et al., 2022; Gnocchi & Bruscalupi, 2017).

Individuals are classified according to their chronotype, such as morningness or eveningness, which can significantly influence performance, mood, and perceived effort. Although findings regarding chronotypes, or individual preferences for morning or evening activity, are less consistent, it is reported that activities that align with an individual's natural circadian rhythm are often associated with improved outcomes (Roenneberg et al., 2007). Differences are most evident in perceived exertion (RPE) rates and mental fatigue, while psychophysiological responses vary across studies (Vitale & Weydahl, 2017). Tasks that require higher cognitive functions may be more influenced by circadian typology, showing a synchrony effect: better performance when tasks are aligned with an individual's circadian rhythm (May & Hasher, 1998; Schmidt et al., 2007). Just as performance levels fluctuate with the time of day, moods are also subject to circadian influences (Irandoust et al., 2019). This raises the possibility that circadian rhythms directly influence mood during physical performance rather than mood changes being merely a consequence of altered performance (Ahmad et al., 2020).

Understanding how these rhythms influence athletic performance and mood can provide valuable insights to optimize training programs to align with natural physiological peaks, ultimately improving overall performance. The primary objective of this study was to investigate the impact of training time (morning vs. evening) on performance, mood, and physiological responses in elite soccer players, with a specific focus on evaluating the influence of training time on technical performance metrics (e.g., ball interceptions, successful passes, and ball possession), physiological responses such as heart rate and lactate levels, and psychological states, including stress, fatigue, and enjoyment, to provide practical insights for coaches to optimize training programs based on athletes' circadian rhythm influences.

## 2 METHODS

### 2.1 Subjects

An a priori sample size calculation was conducted using G*Power version 3.1.9.4 (Heinrich‐Heine‐Universität Düsseldorf, Germany) for a paired‐samples comparison. We assumed a Cohen's d effect size of 0.8 (classified as “large” per Cohen, 1988), with *α* = 0.05 and power (1 – *β*) = 0.80. This yielded a minimum total sample size of *n* = 15 participants. To allow for potential attrition, we enrolled 20 participants, thereby ensuring sufficient power to detect meaningful effects. This approach aligns with prior literature in the field, where similar G*Power‐based estimations were used in small‐sided game interventions with sample sizes ranging from 16 to 24 (Daryanoosh et al., 2023; Pancar et al., 2025).

Twenty male soccer players from the same soccer team competing in the first league (age: 25 ± 0.9 years; height: 1.77 ± 0.07 m; body mass: 72.3 ± 14.2 kg; body fat: 11.6% ± 2.4) participated in the study. The players performed 6–7 regular training sessions per week plus 1 match. The goalkeepers, participating in the same technical and physical training program, were excluded from the study. No player reported injuries during the study. The study was conducted according to the Declaration of Helsinki and approved by the ethics committee of the Higher Institute of Sport and Physical Education of Kef (07/03/2023). Written informed consent was obtained from all participants prior to enrollment in the study.

### 2.2 Procedures

First, anthropometric characteristics and maximum HR (HRmax) were assessed via the standardized VAMEVAL test (Richard et al., 2018). The experimental sessions consisted of 4‐a‐side SSG performed on separate days with a 1‐week interval between sessions. Each participant completed both a morning and an evening SSG session, separated by one week. In each session, participants were randomly split into two subgroups of 10—one performing in the morning, the other in the evening—matched by playing position (3 defenders, 3 defensive midfielders, 4 offensive players). Although each player experienced both time‐of‐day conditions, performance in SSGs inherently depends on interactions with teammates and opponents, who are equally affected by circadian rhythms.

Before each SSG session, oral temperature was measured in the players' seated position for at least 15 min using a calibrated digital clinical thermometer (Omron, Paris, France; accuracy: 0.05°C) by placing it under the tongue for at least 3 min. Each SSG session was 25 min and consisted of 4 bouts of 4 min of gameplay separated by 3 min of passive recovery. HR was monitored throughout each training session.

Video analysis was used to quantify technical actions during SSGs. Players were familiarized with the SSG regimes, TQR scale, and well‐being scales (sleep, stress, fatigue, DOMS) before the beginning of the study. Each experimental session took place in similar conditions of temperature and relative humidity (19°C–33°C and 53%, respectively). Data for each SSG session were registered by the same fitness coach.

### 2.3 Control of sleep, recovery, and chronotype

Throughout the study, players were instructed to maintain consistent sleep patterns and adhere to nutritional guidelines provided by the technical staff to reduce variability in performance outcomes related to external factors. Sleep duration and quality were self‐reported using the Hooper index, which assesses sleep quality on a scale from 1 to 7, with higher scores indicating poorer sleep (Hooper & Mackinnon, 1995; Smith & Doe, 2024). The reported average sleep duration was 7.5 ± 0.8 h per night. While no objective sleep measurements (e.g., actigraphy) were performed, the Hooper index served as a reliable proxy for recovery status. Although individual chronotype preferences were not directly measured, all players were accustomed to both morning and evening training sessions as part of their regular training schedule, minimizing potential bias from unfamiliar training times.

### 2.4 Total quality recovery scale (TQR)

Before each session, to assess the players' recovery state, the TQR scale asked a single question, which was as follows: “What is your condition now?” (Kenttä & Hassmen, 1998).

The TQR scale was measured on a Likert scale and ranging from 6 to 20, where 6 indicates “very, very poor recovery” and 20 indicates “very, very good recovery”, this tool was presented to the participants 15 min before each training session to rate sense of recovery as an overall psychophysiological rating for the past 24 h, including the previous night's sleep. The TQR scale obtained a Cronbach's *α* value of 0.89 in the present study.

### 2.5 Hooper index

Before each session, each player was asked to rate his sleep quality (for the night preceding the training), fatigue, stress, and delayed‐onset muscular soreness (DOMS) using the methods of Hooper (Hooper & Mackinnon, 1995; Selmi et al., 2018).

Each player responded subjectively on a scale of 1–7 with 1 being “very very good” for sleep or “very very low” for the other three indices, and 7 being “very very bad” for sleep and “very very high” for the other indices (Hooper & Mackinnon, 1995). The sum of these four scores (i.e., sleep, fatigue, stress, and DOMS) was summed to calculate the Hooper index (HI). The score of HI ranges from 4 to 28 points. The well‐being indices demonstrated good reliability, with Cronbach's *α* ranging from 0.90 to 0.94 in the present study.

### 2.6 Rating of perceived exertion (RPE)

The internal load was measured at the end of each SSG bout using the CR‐10 RPE scale; (Impellizzeri et al., 2004).

### 2.7 Physiological measures

During the SSGs, the HR was measured via portable HR sensors (Polar Team Sport System, Polar‐Electro OY, Kempele, Finland). For each SSG performed, the average HR from each bout was used to calculate the overall mean HR (HRmean) for the session. The %HRmax for each form of SSG was calculated using the following formula: %HRmax = (HRmean/HRmax) × 100.

### 2.8 VAMEVAL test

During the SSG, HRmax was determined by the VAMEVAL test, conducted on a 200 m running track; participants were asked to perform at maximum effort (Richard et al., 2018). The testing area was prepared using ten cones positioned every 20 m at predetermined locations on the field, guided by a programmed auditory cue (i.e., a beep). The initial running pace was set at 8 km/h and progressively increased by 0.5 km/h every minute until exhaustion. The test took place on a natural grass surface. It was terminated when a participant was unable to sustain the required pace for two consecutive beeps or felt unable to complete the stage (Selmi et al., 2023). Heart rate (HR) was monitored using a PolarTeam Sport System (Polar‐Electro OY, Kempele, Finland). The highest 5‐s average HR during the VAMEVAL test was recorded as VAMEVAL‐HRmax. The VAMEVAL protocol's reliability in determining maximal HR has been previously validated, showing a Cronbach's *α* of 0.83 (Buchheit & Laursen, 2013).

### 2.9 Physical enjoyment

Physical enjoyments were measured after the end of each SSG, by the 18‐item Physical Activity Enjoyment Scale (PACES) (Selmi et al., 2022). The players were asked to quantify “how you feel at the moment regarding the SSG you experienced” by a 7‐points scale (ranging from 1, it's very enjoyable to 7, it's not fun at all). The score ranges from 18 to126 points. The Cronbach's *α* value of the physical enjoyment test was 0.89 in the present study.

### 2.10 Small‐sided games

The SSGs (4‐a‐side) were performed on an outdoor pitch with natural grass on a playing surface (35 × 25 m [~109 m2 per player]). The SSG duration and the pitch were strictly controlled at 4 bouts of 4 min each with 3 min of passive recovery between bouts for a total of 25 min, as reported previously (Selmi et al., 2023). The players were asked to perform at maximum effort during the games and to maintain possession of the ball for the longest possible time. SSGs were played without a goalkeeper. The number of ball touches authorized per individual possession was fixed at two touches to ensure all participants engaged in the SSG. During the SSG, 2 coaches were positioned around the pitch to provide new balls when necessary to allow continuity of the play during the sessions and encourage the players.

Each SSG was preceded by a standardized 20‐min warm‐up, which consisted of 15 min of athletics exercises (i.e., jogging, coordination movements, and dynamic stretching, and ended with 4 × 10‐m sprints with direction changes) and 5 min of simple technical–tactical tasks. Three minutes of recovery separated the warm‐up from the SSG bout. Players were allowed to consume drinks after the warm‐up and during the SSG recovery periods.

### 2.11 Technical performance

The training was filmed using a video camera (Sony Handycam DVD 850, Sony Inc., Tokyo, Japan) to determine technical aspects completed during the entire duration of the SSGs. The technical aspects measured after performing each SSG were as follows: number of individual interceptions, number of individual lost balls, percentage of individual successful passes, number of individual ball possessions, number of individual tackles, number of individual duels, and number of individual headers (Selmi et al., 2023). Although the possession is frequently a team‐level measure, in our crossover design, it reflects individual technical and cognitive performance, particularly when interpreted alongside technical (interceptions, passes) data. Video cameras were placed in an elevated position at the halfway line of each pitch, set 10 m behind the side line. This method has been described as a reliable evaluation of technical action in soccer; the technical variables were chosen because each was specific to soccer, each has been previously defined and examined together in soccer literature, and each was performed repeatedly during SSGs and soccer matches. The same expert researcher reviewed all SSG videos. Each was examined twice to assess intrareader reliability using the kappa coefficient (Kelly & Drust, 2009). The kappa values for the analyzed variables ranged from 0.86 to 0.95, showing a high level of intrareader reliability.

### 2.12 Statistical analyses

Results were analyzed by GraphPad PRISM 5 software. First, all variables were checked for the normality of distribution by the Kolmogorov–Smirnov test. Student's paired *t*‐test was used; *p* < 0.05 was accepted as a level of statistical significance. Effect sizes were reported as Cohen's D.

## 3 RESULTS

### 3.1 Internal load responses

**FIGURE 1:**
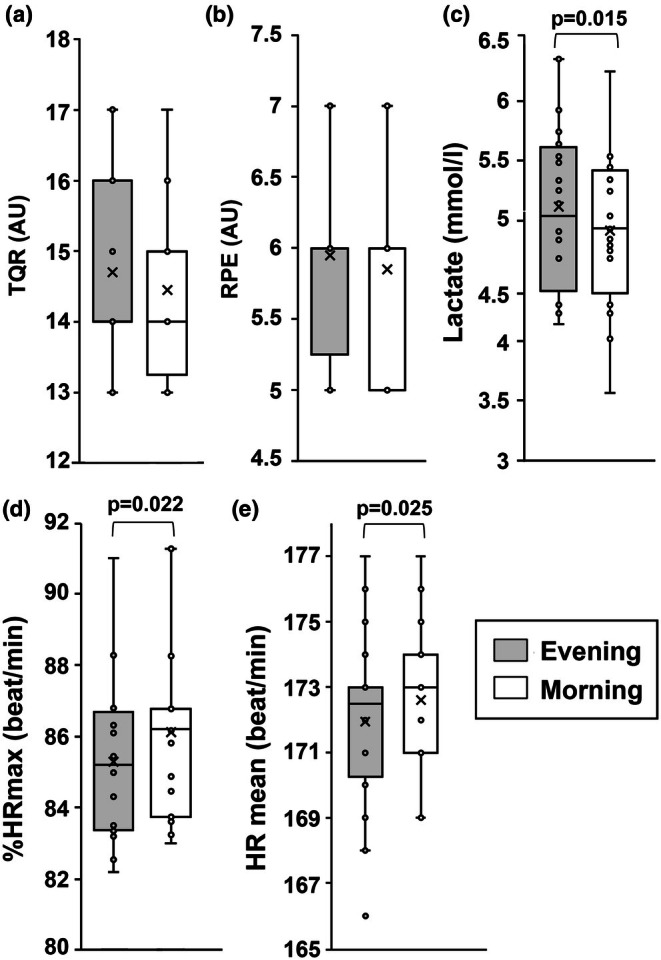
Total quality recovery (TOR) (a), Rating of perceived exertion (RPE) (b), Lactate concentration (C), maximal heart rate (%HRmax) (d) and average heart rate (HRmean), (e) measured during training for small‐sided games training performed in the morning or evening. In this representation, the central box covers the middle 50% of the data values, between the upper and lower quartiles. The bars extend to the extremes; the central line represents the median, and the cross indicates the mean value. The individual points are also shown. *p* value by paired student *t*‐test.

Total quality recovery (TOR) and RPE values were consistent across sessions, showing no significant differences. However, HRmean and %HRmax were higher in the morning (*p* = 0.025, and *p* = 0.022, respectively; Figure 1e,d and respectively). Additionally, a significantly lower [La] concentration was observed in the morning (*p* = 0.015, Figure 1c).

### 3.2 The profile of mood state

**FIGURE 2:**
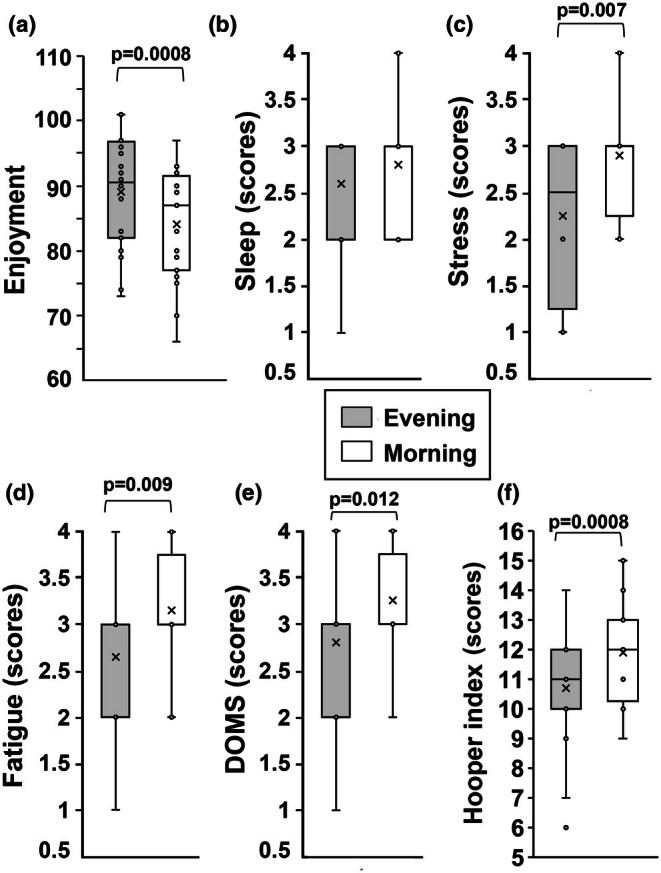
Enjoyment (a) and Hooper index scores (b–f) which include four subjective assessments: sleep, stress, fatigue, and delayed‐onset muscle soreness (DOMS), measured during training for small‐sided games performed in the morning or evening. In this representation, the central box covers the middle 50% of the data values, between the upper and lower quartiles. The bars extend to the extremes, the central line represents the median, and the cross indicates the mean value. The individual points are also shown. *p* value by paired Student *t*‐test.

Players showed greater enjoyment in small‐team games in the evening (*p* = 0.0008, g = 0.63, medium‐to‐large effect; Figure 2a).

In addition, the results showed significantly elevated levels of stress (*p* = 0.007, *g* = 0.926, large effect), fatigue (*p* = 0.009, *g* = 0.704, large effect), DOMS (*p* = 0.012, *g* = **0.533, moderate effect**), and Hooper's index (*p* = 0.0008, *g* = 4.267, a very large effect) in the morning, compared to the evening (Figure 2).

### 3.3 Performance assessment

**FIGURE 3:**
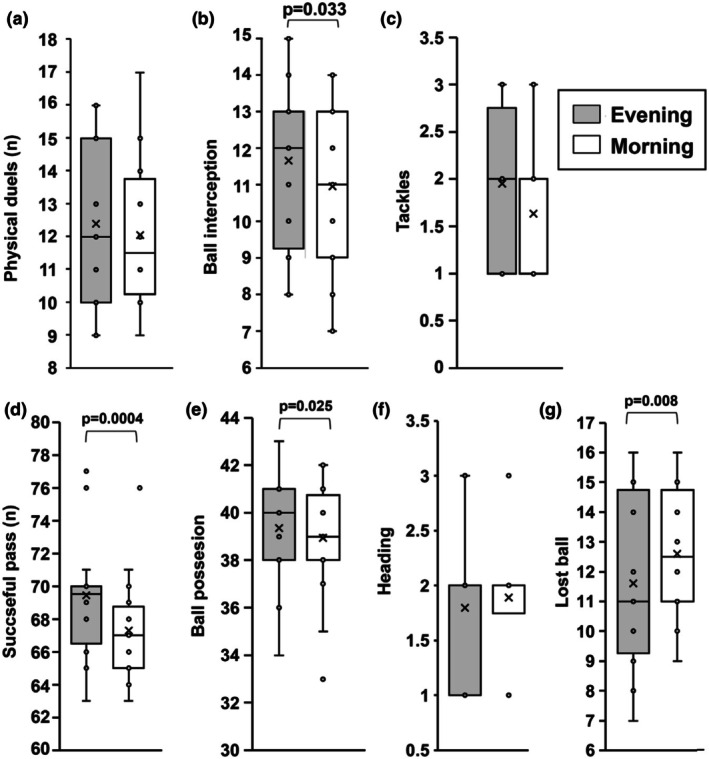
Technical aspects which included: number of duels (a), individual ball interceptions (b), individual tackles (c), number of individual duels (d), percentage of individual successful passes (e), number of individual headers (f) and lost balls (g), measured during each SSG performed in the morning or in the evening. In this representation, the central box covers the middle 50% of the data values, between the upper and lower quartiles. The bars extend to the extremes, the central line represents the median, and the cross indicates the mean value. The individual points are also shown. *p* value by paired Student *t*‐test.

The results showed significantly better performance in terms of ball interceptions (*p* = 0.033; *g* = 0.3, small effect) successful passes (*p* = 0.0004; *g* = 0.7 large effect), and ball possession interceptions (*p* < 0.025; *g* = 0.3, small effect) was observed in the evening (Figure 3), reflecting peak cognitive and physical performance during this time (Halson, 2014; Kline et al., 2010).

Consistently, a significantly higher number of lost balls was observed during morning training (*p* = 0.008; *g* = 0.5, medium effect) (Figure 3g).

Although tackling was higher in the evening and heading was more frequent in the morning, these findings were not statistically significant. The variations might be due to individual differences or specific game contexts rather than a clear circadian effect.

## 4 DISCUSSION

Understanding the influence of circadian rhythms on athletic performance, mood, and physiological responses is crucial for optimizing training and daily activities. Circadian rhythms regulate various physiological processes, including heart rate, metabolic activity, and cognitive function, which can significantly affect exercise outcomes and overall well‐being.

Our study hypothesized that training at different times of day could significantly influence players' performance and recovery, with potential benefits in scheduling sessions according to natural circadian rhythms. Thus, we aimed to determine how training at different times of day affects soccer players' performance, mood, and physiological responses. It focused on whether morning or evening sessions influence metrics like ball interceptions, heart rate, and lactate levels, as well as players' stress, fatigue, recovery, and enjoyment. By examining these factors, we can gain insights into how timing and circadian patterns affect athletic performance and overall enjoyment; the goal was to help coaches and athletes optimize training by aligning it with natural circadian rhythms for better performance and well‐being.

Our observation that both HRmean and HRmax were notably higher in the morning can be linked to circadian rhythms affecting cardiovascular function. Circadian cycles influence heart rate variability and overall cardiovascular efficiency, with a general trend of higher heart rates during the early hours due to the body's natural cortisol peak, which prepares it for activity by increasing alertness and heart rate (Chtourou & Souissi, 2012; Gnocchi & Bruscalupi, 2017). This morning elevation in heart rate is thought to be part of the body's natural process of gearing up for the day's activities. However, despite the elevated heart rate, this does not necessarily translate to enhanced performance. Physiological arousal and metabolic activity are lower in the morning, which can result in different exercise outcomes compared to later in the day (Atkinson & Reilly, 1996; Wolff & Esser, 2019). The increased heart rate in the morning might also reflect a compensatory mechanism to maintain adequate oxygen delivery during exercise when other physiological processes are not yet at their peak efficiency.

Interestingly, the higher HRmean and HRmax in the morning, along with a significantly lower lactate concentration ([La]), align with circadian rhythm effects on physiological responses. Lower [La] in the morning suggests reduced anaerobic energy contribution at this time (Chtourou, Chaouachi, Hammouda, et al., 2012), which may be due to lower metabolic activity and energy production in the early hours (Chtourou & Souissi, 2012; Gnocchi & Bruscalupi, 2017), which might affect high‐intensity performance.

The circadian influences on lactate concentration and heart rate responses are primarily mediated by the interaction of hormonal rhythms, metabolic efficiency, and cardiovascular regulation. In the morning, higher cortisol levels, which peak shortly after waking, contribute to increased heart rate and alertness (Chtourou & Souissi, 2012; Gnocchi & Bruscalupi, 2017). However, metabolic activity tends to be lower during this time, leading to reduced anaerobic energy contribution and lower lactate production during exercise (Chtourou, Chaouachi, Hammouda, et al., 2012; Hower et al., 2018). In contrast, the late afternoon and evening coincide with peak core body temperature, which enhances enzymatic activity and muscle contractility, promoting greater lactate production and improved performance in anaerobic tasks (Brito et al., 2022; Racinais et al., 2005). Additionally, circadian modulation of autonomic nervous system activity optimizes cardiovascular responses later in the day, resulting in improved efficiency during exercise (Shen et al., 2023; Wolff & Esser, 2019). Understanding these mechanisms underscores the importance of aligning training schedules with circadian peaks to optimize both performance and recovery.

This could suggest that while cardiovascular responses are heightened, overall metabolic efficiency is lower, leading to different exercise adaptations, underscoring the circadian rhythm's role in modulating metabolic processes and highlighting the potential for varying exercise outcomes based on the time of day.

Therefore, the observed absence of significant differences in TQR and RPE across sessions suggests that players' perceptions of exertion and recovery were consistent regardless of the time of day. This may indicate that subjective feelings of effort and recovery are less influenced by circadian rhythms compared to objective physiological measures.

Moods have also been studied at rest and during exercise, and conflicting results have been reported. We reported elevated levels of stress, fatigue, DOMS, and Hooper index in the morning that highlight a more compromised mood and physical state early in the day. This aligns with previous findings suggesting that morning exercise may be less favorable due to higher perceived stress and fatigue (Arciero et al., 2022), since the body's systems are less optimal in the morning (Racinais, 2010; Racinais et al., 2005).

Then, morning exercise often encounters challenges due to “physiological inertia,” due to the body's systems, which are slower to reach peak performance levels compared to the evening. After a night of rest, factors such as muscle viscosity, core temperature, and metabolic efficiency tend to be at a lower point, or “trough” state (Racinais, 2010; Waterhouse et al., 2005). These conditions can make morning exercise, particularly high‐intensity training or testing, feel more strenuous and lead to greater fatigue for individuals.

Conversely, evening sessions might benefit from better mood and lower perceived fatigue, potentially improving performance. In contrast, Maraki et al. (2005) found that morning exercise was associated with higher levels of positive mood, potentially due to increased sunlight exposure. Since sunlight affects mood through its effects on retinal photosensitive ganglion cells and the peri‐thalamic nucleus (Fernandez et al., 2018; Li & Yan, 2020), morning exercise benefits from exposure to natural light, increasing energy and positivity. However, physiological responses to physical activity, varying depending on the time of day, can also influence mood during or after morning exercise and afternoon/evening exercise. For example, melatonin plays a crucial role in regulating the body's circadian rhythm (Chawla et al., 2021; McCarthy et al., 2019) and is closely linked to feelings of tiredness, depression, mood disorders (Kudo et al., 2021; Ogłodek et al., 2016; Tao et al., 2020). Physical activity acts as an external cue that can enhance melatonin secretion (Kruk et al., 2021), and the timing of exercise throughout the day can differently impact melatonin patterns (for instance, evening exercise often results in a delayed melatonin phase (Eastman et al., 1995)), affecting emotional states through various molecular signaling pathways.

The variability in emotional responses to exercise timing, as observed in some studies, may diminish with prolonged training or be influenced by individual circadian rhythms. For instance, a 12‐week jogging intervention showed no significant differences between morning and evening exercise groups, indicating that prolonged exercise might reduce the impact of circadian timing on mood (Lazarus et al., 2015). Furthermore, in depressed individuals, circadian mood fluctuations could mask the effects of exercise timing, leading to less noticeable differences between morning and evening sessions (Rao et al., 2015).

In addition, we found that players experienced higher enjoyment of the small‐sided games in the evening, consistent with previously published research showing enjoyment and performance often peak in the late afternoon or evening (Ainsworth et al., 2020; Halson, 2014). Enjoyment and performance in football are closely linked since enjoyment enhances motivation and reduces perceived exertion, leading to improved focus and effort during training and matches (Xu et al., 2025). In football specifically, enjoyment has been associated with more creative play and greater resilience in the face of challenges, ultimately contributing to better outcomes on the field (Van de Pol et al., 2012).

This is attributed to circadian rhythms optimizing cognitive and physical functions during these times. Elevated body temperature in the late afternoon enhances muscle function and enzyme activity, contributing to better performance (Ainsworth et al., 2020). Cognitive functions, including reaction time and decision‐making, are typically sharper later in the day, enhancing performance in tasks requiring quick thinking and coordination (Halson, 2014; Kline et al., 2010).

Consistent with the afternoon and evening peak in cognitive and physical readiness (Halson, 2014; Kline et al., 2010), we measured performance, such as ball interception, successful passing, and ball possession, to be higher in the evening.

It is well established that exercise performance varies with the time of day, especially the short‐term maximal strength performance, which has been shown to peak in the evening hours (Drust et al., 2005); and also, soccer performances improve in the afternoon as well (Rahnama et al., 2009; Reilly & Edwards, 2007).

Conversely, the increased number of ball losses measured in the morning is consistent with lower performance due to the circadian dip affecting cognitive and physical capabilities (Roenneberg et al., 2007). The trend towards increased tackling in the evening, while not statistically significant, supports the idea of better physical performance during peak circadian times (Chtourou & Souissi, 2012). The increased heading in the morning, although not significant, may reflect specific skills or individual variations rather than a clear circadian effect.

Thus, since circadian rhythms influence physical performance, it is important for coaches and athletes (i.e., soccer players) to consider them as determinants of exercise capacity and performance to achieve the best results in competitions.

The study did not control for other factors that could influence performance and recovery, such as nutrition, sleep quality, or training load. We focused on a specific small‐sided game protocol, which may not fully represent the variety of soccer training or match conditions. A key limitation of the current study is the inability to isolate individual performance effects from the influence of other players, who are themselves equally affected by circadian rhythms. This interdependence in SSG performance metrics introduces potential confounding, as changes in one player's outcomes may reflect collective circadian dynamics rather than purely individual responses. While this limits direct attribution of observed differences to individual physiological rhythms, the design retains ecological validity, reflecting how time‐of‐day effects manifest in realistic team settings. The sample size (20 participants) and the exclusion of goalkeepers reduce the generalizability of the results to other soccer positions or team compositions. Since goalkeepers follow distinct training regimens and performance requirements, the conclusions drawn may not apply to their roles. Furthermore, the small sample size limits the statistical power of the study and the possibility of extending the results to larger populations or different competitive levels. Additionally, individual chronotypic preferences were not directly measured. Since chronotype differences could significantly influence performance, mood, and perceived effort, this lack of measurement could undermine the impact of circadian responses on outcomes. In summary, while the results provide valuable insights into the influence of circadian rhythms on soccer performance and recovery, the limitations identified highlight the need for further research. Future studies should consider larger samples, include measures of chronotype, and evaluate a broader range of training scenarios to improve the applicability and robustness of the conclusions.

## 5 CONCLUSION

The study shows that circadian rhythms influence cardiovascular responses, metabolic activity, and mood. Higher HRmean and HRmax in the morning, coupled with lower lactate concentrations, suggest that while cardiovascular responses are elevated, metabolic efficiency is lower in the early hours of the day. The variability of mood and performance by time of day highlights the importance of considering individual circadian patterns to optimize training and athletic performance.

As circadian rhythms play a significant role in physical and sports performance, influencing both physiological and psychological factors, by aligning training and competition programs to these natural rhythms, athletes can potentially optimize their performance. However, individual differences in chronotype and the complex relationship between circadian rhythms and cognitive performance suggest that personalized approaches may be needed to fully exploit the benefits of circadian alignment in sports (Zerbini & Merrow, 2017). As research in this field continues to evolve, it promises to improve training methodologies and performance strategies in sports.

## CONFLICT OF INTEREST STATEMENT

The authors declare that they have no known competing financial interests or personal relationships that could have appeared to influence the work reported in this paper.

## ETHICS STATEMENT

The study was conducted according to the Declaration of Helsinki and approved by the ethics committee of the Higher Institute of Sport and Physical Education of Kef (07/03/2023). Written informed consent was obtained from all participants prior to inclusion in the study.

## Data Availability

Data will be made available on request.
